# Implementing competency based admissions at the Albert Einstein College of Medicine

**DOI:** 10.3402/meo.v21.30000

**Published:** 2016-02-03

**Authors:** Noreen Kerrigan, Myles H. Akabas, Thomas F. Betzler, Maria Castaldi, Mary S. Kelly, Adam S. Levy, Michael J. Reichgott, Louise Ruberman, Siobhan M. Dolan

**Affiliations:** 1 Office of Admissions, Albert Einstein College of Medicine, Bronx, NY, USA; 2 Department of Physiology & Biophysics, Albert Einstein College of Medicine, Bronx, NY, USA; 3 Medical Scientist Training Program, Albert Einstein College of Medicine, Bronx, NY, USA; 4 Department of Psychiatry and Behavioral Sciences, Montefiore Behavioral Health Center at Westchester Square, Bronx, NY, USA; 5 Department of Surgery, Albert Einstein College of Medicine, Jacobi Medical Center, Bronx, NY, USA; 6 Department of Psychiatry and Behavioral Sciences, Albert Einstein College of Medicine, Bronx, NY, USA; 7 Office of Academic Support and Counseling, Albert Einstein College of Medicine, Bronx, NY, USA; 8 Clinical Pediatrics (Hematology & Oncology), Montefiore Medical Center, Albert Einstein College of Medicine, Bronx, NY, USA; 9 Department of Medicine, Albert Einstein College of Medicine, Bronx, NY, USA; 10 Conflict of Interest Committee, Albert Einstein College of Medicine, Bronx, NY, USA; 11 Department of Psychiatry and Behavioral Sciences, Montefiore Medical Center, Albert Einstein College of Medicine, Bronx, NY, USA; 12 Child and Adolescent Psychiatry, Montefiore Medical Center, Albert Einstein College of Medicine, Bronx, NY, USA; 13 Department of Obstetrics & Gynecology and Women's Health, Montefiore Medical Center, Albert Einstein College of Medicine, Bronx, NY, USA

**Keywords:** diversity, competency, admissions, access, community service

## Abstract

The Albert Einstein College of Medicine (Einstein) was founded in 1955 during an era of limited access to medical school for women, racial minorities, and many religious and ethnic groups. Located in the Bronx, NY, Einstein seeks to educate physicians in an environment of state-of-the-art scientific inquiry while simultaneously fulfilling a deep commitment to serve its community by providing the highest quality clinical care. A founding principle of Einstein, the basis upon which Professor Einstein agreed to allow the use of his name, was that admission to the student body would be based entirely on merit. To accomplish this, Einstein has long used a ‘holistic’ approach to the evaluation of its applicants, actively seeking a diverse student body. More recently, in order to improve its ability to identify students with the potential to be outstanding physicians, who will both advance medical knowledge and serve the pressing health needs of a diverse community, the Committee on Admissions reexamined and restructured the requirements for admission. These have now been categorized as four ‘Admissions Competencies’ that an applicant must demonstrate. They include: 1) cocurricular activities and relevant experiences; 2) communication skills; 3) personal and professional development; and 4) knowledge. The purpose of this article is to describe the process that resulted in the introduction and implementation of this competency based approach to the admission process.

Albert Einstein College of Medicine (Einstein) was founded during an era of limited access to medical school for women, racial minorities, and many religious and ethnic groups. Einstein is situated in the Bronx, NY, an ethnically diverse, economically disadvantaged, and medically underserved, inner city region. The Bronx is comprised of more than 1.4 million residents, 43.3% of whom identify themselves as Black or African American and 54.6% who report some Hispanic or Latino ancestry (1).

Einstein seeks to educate physicians in state-of-the art scientific inquiry while simultaneously teaching them to provide the highest quality clinical care. The school's motto, ‘Science at the Heart of Medicine’, exemplifies this dual responsibility (2). Equally important is our commitment to serving the diverse Bronx community, a commitment that will be enhanced by increasing the number of underrepresented minority medical students who train in our institution (3).

Thus, assuring equitable access and creating an educational environment characterized by diversity are core principles for Einstein, and as our mission statement suggests, ‘… we welcome students, faculty, and staff from diverse backgrounds who strive to enhance human health in the community and beyond (2)’.

To accomplish this, the Committee on Admissions has long used holistic review (4), which considers a number of factors that may predict that an applicant has the potential to be an excellent physician, rather than focusing primarily on traditional academic metrics. The committee understands the educational benefits of a diverse learning environment, the social justice imperative to offer students of all backgrounds the opportunity to become physicians, and the workforce demand to educate physicians representative of the diverse populations they will be serving (5).

More recently, based upon the call to action from the Association of American Medical Colleges (AAMC) (6), Einstein became an early adopter of the Competency Based Admissions (CBA) approach and initiated a process of transition to CBA. A full-day *Holistic Review in Admissions* workshop was held on site in September, 2011. This resulted in revision of the Admissions Committee mission statement to read as follows:

The Albert Einstein College of Medicine strives to matriculate a diverse group of outstanding students whose academic accomplishments, clinical experiences, community service, and research indicate that they will become exceptional healers, educators, colleagues, patient advocates, scientists, role models, and life-long learners. We are committed to identifying individuals who already have demonstrated the qualities of compassion, empathy, kindness, creativity, professionalism, leadership, and maturity. A diverse student body is consistent with the history and mission of Einstein and supports a key educational objective to raise the cultural awareness and competence of our graduates (2).

Subsequently, using an iterative process including feedback from students, faculty members, the Office of Student Affairs, and the Medical Education Committee, the Admissions Committee defined four major competencies that successful applicants must demonstrate. They are:

1. Cocurricular activities and relevant experiences
2. Communication skills
3. Personal and professional development
4. Knowledge

A specific decision was made to list these competencies with activities and experiences first, so applicants understand the importance of demonstrating motivation and commitment to service. Communication skills and personal and professional development follow next because the ability to communicate clearly, to appreciate the diverse community in which we live and practice, to display cultural sensitivity, to be a team player, and to exhibit leadership are all essential for success as a physician. Knowledge, as assessed by traditional metrics that include grade-point average (GPA) and the Medical College Admission Test (MCAT) comes last. It must be considered but is no longer the single most important determinant of merit. To assess the competencies, faculty interviewers are asked to comment specifically on the following in their interview reports: spoken language ability, interpersonal interaction, ability to listen, cultural awareness and sensitivity, advocacy, ability to withstand stressors, and commitment to medicine.

Once the competencies were defined and the new hierarchy established, feedback was solicited from premedical advisors. One wrote, ‘If students take the time to read [the competencies] and process [them], they will understand why their undergraduate education and experiences are necessary to shape their awareness and knowledge, which will make them better physicians, [as] opposed to a “checklist/shopping list” of things they have to ‘check off’ just to get into med school’. Another commented, ‘I love the commitment to diversity in the opening comment as well as the need for the students to develop their own awareness of diversity. It will require maturity on the part of the applicants to negotiate the kind of ‘uncertainty’ that the competencies present’.

The Committee on Admissions posted these competencies and began using CBA for students applying in the 2014–2015 year. An unanticipated outcome of this process has been the identification of three areas of diversity (in addition to ethnic and racial underrepresentation in medicine) that previously had not been considered explicitly. These include economic disadvantage, military service, and Lesbian, Gay, Bisexual, Transgender, and Queer (LGBTQ) self-identification.

Implementing CBA also has been helpful in answering questions posed by potential applicants. When a student with limited clinical experience inquires about applying, we ask them to reframe the question from, ‘Can I apply now?’ to ‘Have I fulfilled the competencies and am I ready for medical school; and if not, how can I use a gap year to fulfill the competencies?’

CBA also provides greater flexibility and reduces barriers to admission. For example, a student can substitute research experience for a science course. This frees up time to pursue interests and activities that allow applicants to develop greater maturity and to affirm their commitment to medicine. In addition, by requiring students to read and reflect on the competencies, students must also consider their applications in entirety. This allows them to assess whether or not they are a good fit for our institution. Einstein is looking for leaders with demonstrated maturity and with a commitment to practicing medicine in a diverse community, not just for those students who can fulfill a list of requirements.

Of note, implementation of CBA does not appear to have an adverse effect on the number of applicants to Einstein, and the matriculation ratio and traditional academic metrics are somewhat higher for the class entering in 2015.

Implementation of CBA is intended to ‘… allow applicants the opportunity to demonstrate the complex personal dimensions that contribute to being a good doctor (7)’. It is based on an appreciation that the elements of ‘merit’ justifying admission, which should be used to meet the expectation of our namesake, must be more broadly defined than the limited set of cognitive capabilities that have traditionally identified applicants as being ready for the academic rigor of medical school. By doing so, we intend to open opportunity to a more diverse student body with the ultimate goal of developing a more diverse cadre of physicians.

Demonstration of the effectiveness of this initiative will require:

- Short-term analysis of the composition of matriculants to Einstein
- Intermediate-term evaluation of the performance of the new student body as they progress through undergraduate medical education
- Long-term tracking of residency and post-residency career choices

The implementation of CBA is but a first step in this process.
